# Benefits of team sport participation versus concerns of chronic traumatic encephalopathy: prioritizing the health of our youth

**DOI:** 10.2217/cnc-2020-0006

**Published:** 2020-05-14

**Authors:** Scott L Zuckerman, Aaron M Yengo-Kahn, Benjamin L Brett, Andrew W Kuhn, Daniel I Wolfson, Zachary Y Kerr

**Affiliations:** 1 Vanderbilt Sports Concussion Center, Vanderbilt University Medical Center, Nashville, TN 37232, USA; 2 Department of Neurological Surgery, Vanderbilt University Medical Center, Nashville, TN 37232, USA; 3 Department of Neurology, Medical College of Wisconsin, Milwaukee, WI 53226, USA; 4 Department of Neurosurgery, Medical College of Wisconsin, Milwaukee, WI 53226, USA; 5 Department of Exercise & Sport Science, University of North Carolina at Chapel Hill, Chapel Hill, NC 27154, USA; 6 Matthew Gfeller Sport-Related Traumatic Brain Injury Research Center, University of North Carolina, Chapel Hill, NC 27154, USA; 7 Injury Prevention Research Center, University of North Carolina, Chapel Hill, NC, USA

**Keywords:** chronic traumatic encephalopathy, CTE, neurodegeneration, sports, team sport participation, traumatic brain injury

Over the last decade, discussion of chronic traumatic encephalopathy (CTE) and the sport of American football have become inseparable. The presumed risk of CTE and resultant neurodegenerative changes from contact sport participation has been propelled into the minds of concerned parents and players at all levels. Currently, the literature is mixed on whether contact sport participation is associated with adverse long-term neurologic outcomes, including neurobehavioral changes such as depression and a range of neurodegenerative diseases (dementia, Alzheimer’s disease, etc.) [1–8]. Further, some suggest that the media may give disproportionate attention to research promoting a link between contact sport participation and adverse long-term neurologic outcomes [9].

It has been argued that the linkage of contact sport participation and adverse long-term neurologic outcomes has been prematurely reached without adequate assessment of the true degree of association [10,11]. The public and media may be unaware of the scientific controversy, as media reports sometimes fail to articulate the underlying strengths and limitations of published research [12]. Stewart and 60 experienced traumatic brain injury researchers emphasized concern that the many uncertainties surrounding CTE may lead individuals to mistakenly attribute treatable neurobehavioral and psychiatric disorders to CTE, an incurable disease unable to be diagnosed definitively in living individuals [13]. These experts have advocated that a ‘first, do no harm’ philosophy is foremost and takes precedence over the need to resolve knowledge gaps surrounding CTE. Moreover, some have maintained that we must not only consider those cases that are considered ‘false positives’ (i.e., those who believe they have CTE, but in reality do not), but also the possible ‘false negatives’ (i.e., those who do not believe they have CTE, but may in fact do) [14].

Public concern has manifested in two ways. First, participation in youth and adolescent team sports has declined [15]. Second, efforts have been made to pass legislation that bans youth tackle football [16]. Legal interventions are created, passed and implemented with good intentions, yet they may cause unintended consequences [16], such as exacerbating the already declining physical and mental health of our youth [17].

We continue to encourage cordial dialogue about the potential association between contact sport participation and adverse long-term neurologic outcomes, with a focus on making informed decisions weighted in science and without overextension of what the research cannot tell us. Further, discussion of the ‘first, do no harm’ philosophy must consider numerous factors beyond both the diagnosis/misdiagnosis of CTE and purported adverse long-term neurologic outcomes associated with contact sport participation. We are concerned that clinicians may be inflicting unnecessary harm by discouraging healthful athletic activity through team sport participation altogether. A recent survey of pediatricians revealed that 52% would not allow their child to play contact sports, which is particularly concerning given the absence of reliable studies showing associations between youth contact sport participation and adverse long-term neurologic outcomes [18]. Based on this notion, our commentary aims to: 1) summarize the concerns related to reduced team sport participation and declining physical activity in our youth; 2) describe the benefits of team sports; and 3) offer recommendations regarding team sport participation that address both concerns over long-term adverse neurologic outcomes and our collective role to ensure safe sport settings.

## Declining youth team sport participation & health

For the first time in 30 years, the National Federation of State High School Associations (IN, USA) reported a decline in the number of high school students participating in school-sanctioned sports; in 2018–19, there were 43,495 fewer high school student-athletes compared with the year prior [15]. The two largest contributors to the decline were in football and basketball, with football at its lowest mark since the 1999–2000 season. The Aspen Institute (DC, USA) noted that a decade ago, 45% of children (aged 6–12 years) participated in a team sport, compared with 37% in 2020, with the biggest declines in football, ice hockey and soccer [19].

Concomitant with declines in team sport participation, the physical health of our youth has waned [17,20–25]. Recent projections suggest that 10 years from now, one of every two adults will be obese and one in four will be severely obese [26]. Simulation studies suggest that young adults who finish high school severely obese have a 95% chance of remaining that way through adulthood [17], substantially increasing the risk for cardiovascular disease and metabolic syndrome [22]. Although a balanced diet is essential, vigorous physical activity is also a strong predictor of metabolic health [23,25].

Trends in the mental health of our youth warrant equal concern. Each year, nearly one in ten young adults experiences a major depressive event, with rates steadily increasing [27]. In particular, a strong relationship has been found between increased depressive symptoms and increased leisure screen time [20]. Screen time and video games have both been positively correlated with increased BMI in a study of 2930 adolescents [28]. Screen time not only displaces physical activity but increases energy intake in the form of high calorie snacks, drinks and fast food [29,30]. Excessive digital media use by youth and adolescents has also been suggested to hinder social coping, a focused mind and psychophysiological resilience [31]. Additionally, preliminary evidence has suggested that higher screen-based media use in younger children is associated with poorer literacy skill development and decreased microstructural integrity in select white matter tracts [32].

## Benefits of team sports

Among potential activities that promote youth and adolescent health [33], team sport participation influences all dimensions of physical, mental and social development [34]. Adolescents who participate in organized sports are more likely to meet physical activity standards [21], excel at cardiovascular fitness and endurance [35] and demonstrate lower body fat percentage [36]. Psychosocially, organized sport participation has been positively associated with improved social competence [37] and receiving more social support from friends and family [36]. A study of over 10,000 middle- and high-school athletes showed that physically active adolescents and team sport participants had higher self-esteem, higher life satisfaction and decreased psychological distress [38]. A prospective study of over 1322 collegiate athletes concluded that increased physical activity and sport participation were associated with higher mental health scores [39]. As concluded by a systematic review, team sport participation promotes improved social and psychological health [24].

The field of medicine similarly touts the benefit of prior team sport experience. Otolaryngology residency applicants who excelled in team sports had higher faculty ratings throughout training [40], and team sport participation was associated with selection of chief resident status among radiologists [41]. Orthopedic surgery applicants with collegiate sport participation portrayed increased grit, perseverance and self-control throughout residency [42]. An additional study also found that orthopedic and neurosurgical department chairs played high school or collegiate football more often than the general population [43].

Communication, emotional intelligence and self-discipline are necessary skills for a leader, and all can be ingrained through team sports [44]. Behavioral studies have found that successful teams model shared leadership [45–47], indicating that all athletes on a team can and should develop leadership abilities regardless of ‘captain’ status. Be it a football team or crew boat, team sports teach accountability and reinforce the value of individuals working together in specific roles rather than attempting to ‘go it alone’ [46]. Few other activities during the formative years of young adulthood can impart these powerful life lessons.

Although there are numerous benefits of team sports participation, it should be said that individual sports (i.e., tennis, golf) may also reinforce certain leadership and perseverance qualities, albeit without the team-play aspect. It is also important to recognize that team sports cover the gamut of contact levels, from noncontact (i.e., cross country, swimming) to limited contact (i.e., baseball/softball, volleyball) to contact (i.e., football, soccer) [48]. With that said, options for sport may vary based on situational, personal and community factors. Given that children’s confidence and self-esteem have been associated with their decision to participate in physical activity/sport [49], limiting sport options may result in decreased participation [34]. Moreover, if few sports are available, an athlete may be forced to specialize early, which has been associated with overuse injuries and burnout [34,50–52]. Thus, it is important that as we argue for the benefits of team sport participation, we acknowledge the potential limitations in its availability and advocate for the equitable creation of a variety of team sport opportunities for boys and girls.

## A path forward

Parents, coaches, peers, teachers, school administrators, physicians, scientists and the media all play significant roles in shaping the youth sport experience [53]. Although there is still debate about the knowledge surrounding CTE and the risk of adverse long-term neurologic outcomes, there should nonetheless be a continued emphasis on creating positive experiences for youth sport participants [1–8,54,55]. We offer the following recommendations that consider both the benefit of team sport participation and the concerns over potential long-term neurologic consequences. The five recommendations below start granularly at the athlete level and expand thereafter to parents, community, stakeholders and society.

1. *Children that receive appropriate concussion management should not discontinue sports due to fear of long-term neurologic consequences.* Physical activity and team sport participation in combination with safe concussion management practices (immediate removal from play, prompt diagnosis and individualized recovery led by a knowledgeable healthcare professional) should be emphasized. Empowering and encouraging athletes without contraindications and no active concussion symptoms to continue the sport that is naturally most attractive to them fosters a positive, memorable and lasting team sport experience.
2. *Parents should maximize the benefits of safe team sport participation to their children.* Parents play a significant role in initiating childhood sport participation and maintaining it [44]. Active parents lead to active children [56,57], and having both parents and the entire family involved in physical activity has been associated with increased number of sports played and more sports practiced per week [57]. Parents are also encouraged to proactively ask coaches and administrators about the safety and preventive measures enacted in their children’s sports leagues.
3. *Encouraging team sport and physical activity in children is a multitiered effort that involves local communities.* Substantial evidence exists relating sedentary lifestyle choices to poor health outcomes [20,22,25,28–31], while little evidence exists demonstrating significant long-term risks of team sport participation in a safe setting [1–8]. Communities are encouraged to develop and support a variety of youth sport participation opportunities, such as recreational and travel leagues that allow youth to choose the team sport most attractive to them [58]. However, these sport opportunities must embrace a culture of safety that includes injury prevention and appropriate management for all potential injuries [58].
4. *To better promote team sport participation, sports safety must include stakeholder buy-in, feasibility considerations and scientific evaluation.* We encourage the development of rule variants, equipment changes and other creative approaches that promote safety and team sport participation. The scientific evaluation of these safety measures is critical to ensure they achieve their stated objective and are implemented appropriately.
5. *Continue to bridge the disconnect between science and the media.* The public should be well-informed about the benefits and risks of team sport participation stratified by overall sport and contact level. A collaborative relationship between scientists and the media is needed to ensure accurate information is disseminated. While strategies to bridge the disconnect have been previously recommended [59], it is important to seek guidance from both parties. Scientists and the media must maintain objectivity, strive for transparency and attempt to reduce biases and personal positions in pursuit of a common goal – health and safety in sport.

## Conclusion

Team sport participation is an integral ingredient in promoting the physical and mental health development of our youth. The ‘first, do no harm’ approach of Stewart *et al.* [13], upon which we have expounded, blends three important notions that: first, we recognize that little evidence demonstrates significant adverse neurologic risk of safe team sport participation; second, team sport participation has an unequivocally positive impact on youth; and third, a prerequisite to any sport is a culture that prioritizes the health, safety and well-being of its athletes [60].

### Endorsements:

The following researchers and medical professionals support and endorse the conclusions formed in this commentary:

- John Amburgy (University of Alabama-Birmingham)
- Julian E. Bailes (University of Chicago Pritzker School of Medicine)
- Christopher M. Bonfield (Vanderbilt University Medical Center)
- Dennis A. Cardone (New York University Langone Health)
- Rudolph J. Castellani (West Virginia University)
- Peter Cummings (Veritas Sports Injury Research Network)
- Gavin A. Davis (Cabrini Hospital)
- Katherine Gifford (Vanderbilt University Medical Center)
- Andrew Gregory (Vanderbilt University Medical Center)
- Lili-Naz Hazrati (The Hospital for Sick Children)
- Mark Herceg (Phelps Hospital Northwell Health)
- Grant L. Iverson (Massachusetts General Hospital)
- Aaron S. Jeckell (Vanderbilt University Medical Center)
- Tim Lee (Vanderbilt University Medical Center)
- Joseph C. Maroon (University of Pittsburgh Medical Center)
- Uzma Samadani (University of Minnesota)
- Douglas P. Terry (Harvard Medical School)

## References

[1] Omalu BI, DeKosky ST, Minster RL, Kamboh MI, Hamilton RL, Wecht CH Chronic traumatic encephalopathy in a National Football League player. Neurosurgery 57(1), 128–134 (2005). 10.1227/01.neu.0000163407.92769.ed 15987548

[2] Mez J, Daneshvar DH, Kiernan PT Clinicopathological evaluation of chronic traumatic encephalopathy in players of American football. JAMA 318(4), 360–370 (2017). 28742910 10.1001/jama.2017.8334 PMC5807097

[3] Janssen PH, Mandrekar J, Mielke MM High school football and late-life risk of neurodegenerative syndromes, 1956-1970. Mayo Clin. Proc. 92(1), 66–71 (2017). 27979411 10.1016/j.mayocp.2016.09.004 PMC5452974

[4] Savica R, Parisi JE, Wold LE, Josephs KA, Ahlskog JE High school football and risk of neurodegeneration: a community-based study. Mayo Clin. Proc. 87(4), 335–340 (2012). 22469346 10.1016/j.mayocp.2011.12.016 PMC3538465

[5] Deshpande SK, Hasegawa RB, Rabinowitz AR Association of playing high school football with cognition and mental health later in life. JAMA Neurol. 74(8), 909–918 (2017). 28672325 10.1001/jamaneurol.2017.1317 PMC5710329

[6] Willer BS, Zivadinov R, Haider MN, Miecznikowski JC, Leddy JJ A preliminary study of early-onset dementia of former professional football and hockey players. J. Head Trauma Rehabil. 33(5), E1–E8 (2018). 10.1097/HTR.0000000000000421 PMC6126972 30080796

[7] Willer BS, Tiso MR, Haider MN Evaluation of executive function and mental health in retired contact sport athletes. J. Head Trauma Rehabil. 33(5), E9–E15 (2018). 10.1097/HTR.0000000000000423 PMC6126955 30080797

[8] Lehman EJ, Hein MJ, Baron SL, Gersic CM Neurodegenerative causes of death among retired National Football League players. Neurology 79(19), 1970–1974 (2012). 22955124 10.1212/WNL.0b013e31826daf50 PMC4098841

[9] Wolfson DI, Kuhn AW, Kerr ZY Chronic traumatic encephalopathy research viewed in the public domain: What makes headlines?. Brain Inj. 34(4), 528–534 (2020). 32064946 10.1080/02699052.2020.1725843

[10] Castellani RJ, Perry G, Iverson GL Chronic effects of mild neurotrauma: putting the cart before the horse? J. Neuropathol. Exp. Neurol. 74(6), 493–499 (2015). 25933385 10.1097/NEN.0000000000000193 PMC4433573

[11] Iverson GL, Gardner AJ, McCrory P, Zafonte R, Castellani RJ A critical review of chronic traumatic encephalopathy. Neurosci. Biobehav. Rev. 56, 276–293 (2015). 26183075 10.1016/j.neubiorev.2015.05.008

[12] Merz ZC, Van Patten R, Lace J Current public knowledge pertaining to traumatic brain injury: influence of demographic factors, social trends, and sport concussion experience on the understanding of traumatic brain injury sequelae. Arch. Clin. Neuropsychol. 32(2), 155–167 (2017). 27765734 10.1093/arclin/acw092

[13] Stewart W, Allinson K, Al-Sarraj S Primum non nocere: a call for balance when reporting on CTE. Lancet Neurol. 18(3), 231–233 (2019). 10.1016/S1474-4422(19)30020-1 PMC6594011 30784550

[14] Brand KP, Finkel AM A decision-analytic approach to addressing the evidence about football and chronic traumatic encephalopathy. Semin. Neurol. [Epub ahead of print] (2019). 10.1055/s-0039-1688484 31311037

[15] National Federation of State High School Associations. Participation in high school sports registers first decline in 30 years (2019). www.nfhs.org/articles/participation-in-high-school-sports-registers-first-decline-in-30-years/

[16] USA Today. New York lawmakers are considering a ban on tackle football for kids under 12 (2020). www.usatoday.com/story/news/nation/2019/10/31/new-york-considers-ban-youth-tackle-football-sparks-cte-debate/4107795002/

[17] Ward ZJ, Long MW, Resch SC, Giles CM, Cradock AL, Gortmaker SL Simulation of growth trajectories of childhood obesity into adulthood. N. Engl. J. Med. 377(22), 2145–2153 (2017). 29171811 10.1056/NEJMoa1703860 PMC9036858

[18] Fishman M, Taranto E, Perlman M, Quinlan K, Benjamin HJ, Ross LF Attitudes and counseling practices of pediatricians regarding youth sports participation and concussion risks. J. Pediatr. 184, 19–25 (2017). 28238481 10.1016/j.jpeds.2017.01.048

[19] The Aspen Institute. State of Play: 2018 Trends and Developments. https://assets.aspeninstitute.org/content/uploads/2018/10/StateofPlay2018_v4WEB_2-FINAL.pdf?_ga=2.56912297.2049237436.1587981351-1091643186.1587981351

[20] Hoare E, Milton K, Foster C, Allender S The associations between sedentary behaviour and mental health among adolescents: a systematic review. Int. J. Behav. Nutr. Phys. Act. 13(1), 108 (2016). 27717387 10.1186/s12966-016-0432-4 PMC5055671

[21] Vella SA, Cliff DP, Okely AD, Scully ML, Morley BC Associations between sports participation, adiposity and obesity-related health behaviors in Australian adolescents. Int. J. Behav. Nutr. Phys. Act. 10, 113 (2013). 24088327 10.1186/1479-5868-10-113 PMC3851783

[22] Skinner AC, Perrin EM, Moss LA, Skelton JA Cardiometabolic risks and severity of obesity in children and young adults. N. Engl. J. Med. 373(14), 1307–1317 (2015). 26422721 10.1056/NEJMoa1502821

[23] Aadland E, Kvalheim OM, Anderssen SA, Resaland GK, Andersen LB The multivariate physical activity signature associated with metabolic health in children. Int. J. Behav. Nutr. Phys. Act. 15(1), 77 (2018). 30111365 10.1186/s12966-018-0707-z PMC6094580

[24] Andersen MH, Ottesen L, Thing LF The social and psychological health outcomes of team sport participation in adults: an integrative review of research. Scand. J. Public Health 47(8), 832–850 (2019). 30113260 10.1177/1403494818791405

[25] Chaput JP, Barnes JD, Tremblay MS Thresholds of physical activity associated with obesity by level of sedentary behaviour in children. Pediatr. Obes. 13(7), 450–457 (2018). 29573239 10.1111/ijpo.12276

[26] Ward ZJ, Bleich SN, Cradock AL Projected U.S. state-level prevalence of adult obesity and severe obesity. N. Engl. J. Med. 381(25), 2440–2450 (2019). 31851800 10.1056/NEJMsa1909301

[27] Mojtabai R, Olfson M, Han B National trends in the prevalence and treatment of depression in adolescents and young adults. Pediatrics 138(6), e20161878 (2016). 27940701 10.1542/peds.2016-1878 PMC5127071

[28] Furthner D, Ehrenmueller M, Lanzersdorfer R, Halmerbauer G, Schmitt K, Biebl A Education, school type and screen time were associated with overweight and obesity in 2930 adolescents. Acta Paediatr. 107(3), 517–522 (2018). 29131386 10.1111/apa.14149

[29] Pearson N, Biddle SJ Sedentary behavior and dietary intake in children, adolescents, and adults. A systematic review. Am. J. Prev. Med. 41(2), 178–188 (2011). 21767726 10.1016/j.amepre.2011.05.002

[30] Robinson TN, Banda JA, Hale L Screen media exposure and obesity in children and adolescents. Pediatrics 140(Suppl. 2), S97–S101 (2017). 29093041 10.1542/peds.2016-1758K PMC5769928

[31] Lissak G Adverse physiological and psychological effects of screen time on children and adolescents: literature review and case study. Environ. Res. 164, 149–157 (2018). 29499467 10.1016/j.envres.2018.01.015

[32] Hutton JS, Dudley J, Horowitz-Kraus T, DeWitt T, Holland SK Associations between screen-based media use and brain white matter integrity in preschool-aged children. JAMA Pediatr. 174(1), e193869 (2020). 10.1001/jamapediatrics.2019.3869 PMC6830442 31682712

[33] U.S. Department of Health and Human Services. Physical activity guidelines for Americans 2nd edition. (2018). https://health.gov/sites/default/files/2019-09/Physical_Activity_Guidelines_2nd_edition.pdf

[34] Logan K, Cuff S, Council On Sports Medicine and Fitness. Organized sports for children, preadolescents, and adolescents. Pediatrics 143(6), e20190997 (2019). 10.1542/peds.2019-0997 31110166

[35] Carlisle CC, Weaver RG, Stodden DF, Cattuzzo MT Contribution of organized sport participation to health-related fitness in adolescents. Glob. Pediatr. Health 6, 2333794X19884191 (2019). 10.1177/2333794X19884191 PMC6820164 31696145

[36] Agata K, Monyeki MA Association between sport participation, body composition, physical fitness, and social correlates among adolescents: the PAHL study. Int. J. Environ. Res. Public Health 15(12), E2793 (2018). 30544884 10.3390/ijerph15122793 PMC6313443

[37] Bedard C, Hanna S, Cairney J A longitudinal study of sport participation and perceived social competence in youth. J. Adolesc. Health 66(3), 352–359 (2020). 31732276 10.1016/j.jadohealth.2019.09.017

[38] Guddal MH, Stensland SO, Smastuen MC, Johnsen MB, Zwart JA, Storheim K Physical activity and sport participation among adolescents: associations with mental health in different age groups. Results from the Young-HUNT study: a cross-sectional survey. BMJ Open 9(9), e028555 (2019). 10.1136/bmjopen-2018-028555 PMC6731817 31488476

[39] Snedden TR, Scerpella J, Kliethermes SA Sport and physical activity level impacts health-related quality of life among collegiate students. Am. J. Health Promot. 33(5), 675–682 (2019). 30586999 10.1177/0890117118817715 PMC7213817

[40] Chole RA, Ogden MA Predictors of future success in otolaryngology residency applicants. Arch. Otolaryngol. Head Neck Surg. 138(8), 707–712 (2012). 22911295 10.1001/archoto.2012.1374

[41] Maxfield CM, Grimm LJ The value of numerical USMLE step 1 scores in radiology resident selection. Acad. Radiol. [Epub ahead of print] (2019). 10.1016/j.acra.2019.08.007 31445825

[42] Camp CL, Wang D, Turner NS, Grawe BM, Kogan M, Kelly AM Objective predictors of grit, self-control, and conscientiousness in orthopaedic surgery residency applicants. J. Am. Acad. Orthop. Surg. 27(5), e227–e234 (2019). 30247313 10.5435/JAAOS-D-17-00545

[43] Sone JY, Courtney-Kay Lamb S, Techar K High prevalence of prior contact sports play and concussion among orthopedic and neurosurgical department chairs. J. Neurosurg. Pediatr. 22(1), 1–8 (2018). 10.3171/2018.1.PEDS17640 29701560

[44] Dobosz RP, Beaty LA The relationship between athletic participation and high school students' leadership ability. Adolescence 34(133), 215–220 (1999). 10234379

[45] Fransen K, Vanbeselaere N, De Cuyper B, Vande Broek G, Boen F The myth of the team captain as principal leader: extending the athlete leadership classification within sport teams. J. Sports Sci. 32(14), 1389–1397 (2014). 24660668 10.1080/02640414.2014.891291

[46] Fransen K, Haslam SA, Mallett CJ, Steffens NK, Peters K, Boen F Is perceived athlete leadership quality related to team effectiveness? A comparison of three professional sports teams. J. Sci. Med. Sport 20(8), 800–806 (2017). 28214098 10.1016/j.jsams.2016.11.024

[47] Leo FM, Garcia-Calvo T, Gonzalez-Ponce I, Pulido JJ, Fransen K How many leaders does it take to lead a sports team? The relationship between the number of leaders and the effectiveness of professional sports teams. PLoS ONE 14(6), e0218167 (2019). 31181130 10.1371/journal.pone.0218167 PMC6557507

[48] Rice SG Medical conditions affecting sports participation. Pediatrics 121(4), 841–848 (2008). 18381550 10.1542/peds.2008-0080

[49] Chase MA Children's self-efficacy motivational intentions, and attributions in physical education and sport. Res. Q. Exerc. Sport 72(1), 47–54 (2001). 11253319 10.1080/02701367.2001.10608931

[50] Myer GD, Jayanthi N, Difiori JP Sport specialization, part I: does early sports specialization increase negative outcomes and reduce the opportunity for success in young athletes? Sports Health 7(5), 437–442 (2015). 26502420 10.1177/1941738115598747 PMC4547120

[51] Post EG, Biese KM, Schaefer DA Sport-specific associations of specialization and sex with overuse injury in youth athletes. Sports Health 12(1), 36–42 (2020). 31724908 10.1177/1941738119886855 PMC6931179

[52] Giusti NE, Carder SL, Vopat L Comparing burnout in sport-specializing versus sport-sampling adolescent athletes: a systematic review and meta-analysis. Orthop. J. Sports Med. 8(3), 2325967120907579 (2020). 32166094 10.1177/2325967120907579 PMC7052469

[53] Noonan RJ, Boddy LM, Fairclough SJ, Knowles ZR Write, draw, show, and tell: a child-centred dual methodology to explore perceptions of out-of-school physical activity. BMC Public Health 16, 326 (2016). 27080384 10.1186/s12889-016-3005-1 PMC4832535

[54] Gardner A, Iverson GL, McCrory P Chronic traumatic encephalopathy in sport: a systematic review. Br. J. Sports Med. 48(2), 84–90 (2014). 23803602 10.1136/bjsports-2013-092646

[55] LoBue C, Schaffert J, Cullum CM Chronic traumatic encephalopathy: understanding the facts and debate. Curr. Opin. Psychiatry 33(2), 130–135 (2020). 31895156 10.1097/YCO.0000000000000580

[56] Mackintosh KA, Knowles ZR, Ridgers ND, Fairclough SJ Using formative research to develop CHANGE!: a curriculum-based physical activity promoting intervention. BMC Public Health 11, 831 (2011). 22032540 10.1186/1471-2458-11-831 PMC3214189

[57] Rodrigues D, Padez C, Machado-Rodrigues AM Active parents, active children: the importance of parental organized physical activity in children's extracurricular sport participation. J. Child Health Care 22(1), 159–170 (2018). 29166768 10.1177/1367493517741686

[58] The Aspen Institute. Project Play: The 8 Plays (2020). http://youthreport.projectplay.us/the-8-plays/introduction

[59] Kuhn AW, Yengo-Kahn AM, Kerr ZY, Zuckerman SL Sports concussion research, chronic traumatic encephalopathy and the media: repairing the disconnect. Br. J. Sports Med. 51(24), 1732–1733 (2017). 27587800 10.1136/bjsports-2016-096508

[60] Kerr ZY, Register-Mihalik JK, Haarbauer-Krupa J Using opinion leaders to address intervention gaps in concussion prevention in youth sports: key concepts and foundational theory. Inj. Epidemiol. 5(1), 28 (2018). 29984386 10.1186/s40621-018-0158-7 PMC6035905

